# Assessing stationary distributions derived from chromatin contact maps

**DOI:** 10.1186/s12859-020-3424-y

**Published:** 2020-02-24

**Authors:** Mark R. Segal, Kipper Fletez-Brant

**Affiliations:** 1 Division of Bioinformatics, Department of Epidemiology and Biostatistics, UCSF, 550 16th Street, San Francisco, 94158 CA USA; 2 Computational Biology, 23andMe, Inc., 899 West Evelyn Avenue, Mountain View, 94041 CA USA

**Keywords:** Chromatin conformation capture, Transition probability matrix, Nearest neighbors, 3D genome reconstruction, Normalization

## Abstract

**Background:**

The spatial configuration of chromosomes is essential to various cellular processes, notably gene regulation, while architecture related alterations, such as translocations and gene fusions, are often cancer drivers. Thus, eliciting chromatin conformation is important, yet challenging due to compaction, dynamics and scale. However, a variety of recent assays, in particular Hi-C, have generated new details of chromatin structure, spawning a number of novel biological findings. Many findings have resulted from analyses on the level of native contact data as generated by the assays. Alternatively, reconstruction based approaches often proceed by first converting contact frequencies into distances, then generating a three dimensional (3D) chromatin configuration that best recapitulates these distances. Subsequent analyses can enrich contact level analyses via superposition of genomic attributes on the reconstruction. But, such advantages depend on the accuracy of the reconstruction which, absent gold standards, is inherently difficult to assess. Attempts at accuracy evaluation have relied on simulation and/or FISH imaging that typically features a handful of low resolution probes. While newly advanced multiplexed FISH imaging offers possibilities for refined 3D reconstruction accuracy evaluation, availability of such data is limited due to assay complexity and the resolution thereof is appreciably lower than the reconstructions being assessed. Accordingly, there is demand for new methods of reconstruction accuracy appraisal.

**Results:**

Here we explore the potential of recently proposed stationary distributions, hereafter StatDns, derived from Hi-C contact matrices, to serve as a basis for reconstruction accuracy assessment. Current usage of such StatDns has focussed on the identification of highly interactive regions (HIRs): computationally defined regions of the genome purportedly involved in numerous long-range intra-chromosomal contacts. Consistent identification of HIRs would be informative with respect to inferred 3D architecture since the corresponding regions of the reconstruction would have an elevated number of *k* nearest neighbors (*k*NNs). More generally, we anticipate a monotone decreasing relationship between StatDn values and *k*NN distances. After initially evaluating the reproducibility of StatDns across replicate Hi-C data sets, we use this implied StatDn - *k*NN relationship to gauge the utility of StatDns for reconstruction validation, making recourse to both real and simulated examples.

**Conclusions:**

Our analyses demonstrate that, as constructed, StatDns do *not* provide a suitable measure for assessing the accuracy of 3D genome reconstructions. Whether this is attributable to specific choices surrounding normalization in defining StatDns or to the logic underlying their very formulation remains to be determined.

## Background

The spatial configuration of chromosomes is essential to various cellular processes, notably gene regulation. Conversely, architecture related alterations, such as translocations and gene fusions, are often cancer drivers. Accordingly, eliciting chromatin conformation is important. Such elicitation had been challenging due to chromatin compaction, dynamics and scale. However, the emergence of the suite of chromatin conformation capture assays, in particular Hi-C, generated new details of chromatin structure and spawned a number of subsequent biological findings [2, 9, 10, 18, 23]. Many of these findings have directly resulted from analyses of interaction or contact level data generated by Hi-C assays. Such data, usually obtained from bulk cell populations, record the frequency with which pairs of genomic loci (or bins thereof) are cross-linked, indicating spatial proximity of those loci within the nucleus. A less common Hi-C analysis paradigm proceeds by first converting these contact frequencies into distances, this transformation often invoking inverse power-laws [2, 13, 29, 35, 41]), and then generating a putative three dimensional (3D) reconstruction of the associated chromatin configuration via variants of multi-dimensional scaling (MDS). Such 3D reconstruction has been shown to enrich analyses based solely on the underlying contact map, these deriving, in part, from superposing genomic features. Examples include identifying co-localized genomic landmarks such as early replication origins [6, 37], expression gradients and co-localization of virulence genes in the malaria parasite *Plasmodium falciparum* [2], the impact of spatial organization on double strand break repair [14], and elucidation of ‘3D hotspots’ corresponding to overlaid ChIP-Seq transcription factor maxima, revealing novel regulatory interactions [7].

But, any potential added value in analyses based on 3D reconstruction is conditional on the accuracy of the corresponding reconstruction and, appropriately, many concerns have been expressed regarding such accuracy. Firstly, the very notion of *a* single reconstruction being representative of the large (∼10^6^) cell populations characterizing Hi-C assays is highly simplistic [19]. This issue has prompted reconstruction approaches [13, 33] that produce an ensemble of solutions, intended to capture inter-cell variation. However, whether these collections capture biologic, as opposed to algorithmic, variation is unclear [26, 35]. The recent development of high-throughput *single-cell* Hi-C assays [22, 31] provides an opportunity for systematic investigation of structural variation. Secondly, even at the single-cell level, genome conformation is dynamic with, for instance, obvious changes over the course of the cell cycle, as well as cell type specific. Finally, the lack of 3D chromatin structure gold standards makes accuracy assessment inherently problematic. To address this obstacle several authors have appealed to simulation [16, 20, 34, 35, 41, 42]. In order to deploy real data referents many of the same reconstruction algorithm developers have made recourse to fluorescence *in situ* hybridization (FISH) imaging as a means for gauging the accuracy of competing algorithms and/or tuning parameter settings. This approach proceeds by comparing measured distances between imaged probes with corresponding distances obtained from 3D reconstruction algorithms. These standard FISH-based methods, however, are tenuous due to the limited number of imaged probes (∼2−6, [18, 20, 29]) and the poor resolution thereof, many straddling over 1 megabase.

To improve on these accuracy assessment shortcomings we previously devised methods that centered on two newly devised biotechnologies [28]: (i) multiplex FISH [36] which provides an order of magnitude more probes, each at higher resolution, and hence two orders of magnitude more distances than conventional FISH, and (ii) a proximity-based ligation-free method, genome architecture mapping [3], predicated on sequencing DNA from a large collection of randomly-oriented, thin nuclear cryosections which enables determination of an internal measure of accuracy by evaluating how well the reconstruction conforms to the underlying collection of planar nuclear cryosections. However, these approaches to accuracy assessment have their own limitations. The primary drawback is that each biotechnology is experimentally intensive and, accordingly, has had minimal uptake. The resultant dearth of associated public data profoundly restricts the extent to which these approaches can be applied. Additionally, there is a resolution disparity, with Hi-C data being available at higher resolutions, mandating a coarsening of reconstructions prior to accuracy assessment.

In seeking to devise a more broadly applicable means for reconstruction accuracy assessment we were drawn to the recently proposed (Sobhy et al., [30], hereafter SKLLS) stationary distribution (hereafter StatDn(s)) of a Hi-C matrix and associated highly interactive regions (HIRs): computationally defined regions of the genome purportedly involved in numerous long-range intra-chromosomal contacts. Consistent identification of HIRs would be informative with respect to inferred 3D architecture since the corresponding regions of the reconstruction would have an elevated number of *k* nearest neighbors (*k*NNs) compared with non-highly interacting regions. More generally, we would anticipate a monotone decreasing relationship between StatDn values and *k*NN distances for fixed values of *k*. This posited relationship provides one means for evaluating the potential utility of StatDns, ithe objective of this paper, which is organized as follows. Under Methods we first recapitulate how StatDns are derived, highlighting normalization and interpretation issues, and then detail data sources to be used in the evaluation thereof. The “Results” section showcases StatDn findings with respect to reproducibility across replicate Hi-C data sets, effects of normalization scheme, and performance for 3D reconstruction validation, via assessment of the above monotonicity between StatDn values and *k*NN distances, based on real and simulated examples. The Discussion frames conclusions based on the foregoing findings.

## Methods

### Stationary distributions from Hi-C contact matrices

Given a (possibly normalized – see below) symmetric, non-negative *n*×*n* observed contact matrix *O*=[*o*_*ij*_] the associated StatDn is generated as follows. First, *O* is standardized by dividing every entry by its row sum. This enables the key step: treating the resultant matrix, *W*, as a transition probability matrix (TPM), with entry *w*_*ij*_ interpreted as the probability of ‘jumping’ from node *i* to node *j* where ‘nodes’ denote a rebranding of the underlying Hi-C bins or loci, thereby allowing an overlay of graph / network concepts. The fact that, due to row sum based standardization, *W* is not symmetric complicates this interpretation since the original ‘proximities’ as measured via Hi-C are symmetric: *o*_*ij*_=*o*_*ji*_. SKLLS proceed by prescribing a Markov model with TPM *W*. Let *p*_*i*_(*t*) be the probability of occupying node *i* at time *t* and *p*(*t*)=(*p*_1_(*t*),*p*_2_(*t*),…,*p*_*n*_(*t*)) be the corresponding probability distribution. Then, under the Markov assumption, transitions occur according to

1 $$ p(t+1) = p(t) W  $$

The limiting (*t*→*∞*) StatDn, designated *p*(*∞*), satisfies *p*(*∞*)=*p*(*∞*)*W*, and is given by the (left) eigenvector corresponding to the (largest) eigenvalue one, the non-negative entries of *p*(*∞*) being normalized to sum to one. We use the R package RSpectra [21] to perform the requisite spectral decomposition.

SKLLS categorize StatDns, at 30^*th*^, 50^*th*^, 80^*th*^ and 90^*th*^ percentiles, and deploy the resultant ordered categories in downstream analyses, with an emphasis on HIRs corresponding to the latter upper decile. In contrast, we utilize StatDns in their native, continuous form obviating the need for thresholding. As a check, we extracted SKLLS-defined categories and reprised select analyses with concordant findings.

### Normalization and interpretation issues

There has been extensive discussion surrounding normalization issues for Hi-C data and development of companion corrective methods [8, 11, 12, 17, 38]. Much of this effort pertains to mitigating systematic biases affecting observed *o*_*ij*_ values deriving from factors such as fragment length, GC content and mappability. A distinct aspect of some normalization strategies concerns removing ‘expected’ contact counts from the observed values so as to adjust for contiguity and thereby emphasize features of interest such as loops. In this context expected values are often computed as a function of genomic distance [2, 10]. This equates to applying a common correction within each diagonal of *O*, elements thereof being equi-spaced with respect to genomic distance, presuming equal sized contact matrix bins as is standard. It is this approach that is considered by SKLLS.

Specifically, for each of the *n* diagonals of *O*, the median of the corresponding entries is obtained. An *n*×*n* expectation matrix *E* with constant diagonals is then created, the constants being the respective medians. In addition to obtaining StatDns (as detailed above) from (unnormalized) *O*, they are also generated from *O*−*E* and *O*/*E*. To satisfy the non-negativity requirement of a TPM any negative values arising post normalization are replaced with a small positive constant. For *O*−*E* normalization, with *E* based on diagonal medians, this means that approximately half the entries will be replaced by this constant. The ramifications, both interpretive and performance-wise, of such wholesale substitution are unclear.

In order to decide between the competing normalization schemes SKLLS assert that *O*−*E* normalization produces StatDns with a larger ‘dynamic range’ than *O* or *O*/*E* approaches, and is accordingly preferred. Presuming dynamic range is defined as the difference between maximum and minimum StatDn values, the rationale for its selection as a normalization criterion is obscure. Moreover, it will be susceptible to the influence of outliers as can arise from extreme (normalized) contact matrix row sums. The supporting evidence presented for choosing *O*−*E* consists of visually comparing StatDns from the three schemes over a limited range of a single chromosome. Further, it is claimed that, in using *O* directly, the inclusion of both short- and long-range contacts attenuates dynamic range but the basis for this is unclear.

It is pertinent to consider StatDns, as operationalized above, arising from specific patterned matrices. For a compound symmetric (exchangeable) matrix the StatDn is constant (*p*_*i*_(*∞*)=1/*n* ∀*i*) irrespective of the value of the off-diagonal entries, with this same StatDn resulting from a tri-diagonal matrix, again independent of the value of the off-diagonal entries [25]. While these patterns don’t reflect *O*,*O*−*E*,*O*/*E* matrices arising in practice, the lack of StatDn discrimination between such appreciably different matrices raises interpretative concerns about the proposed approach, at least from the perspective of evaluating 3D reconstructions, and potentially beyond.

### Data sources and simulated 3D structures

Hi-C data [23] for GM12878 cells was obtained from the Gene Expression Omnibus (GEO) with accession GSE63525. Contact matrices deriving from several series of experiments were grouped (by the original authors) into ‘primary’ and ‘replicate’ datasets and we utilize these to assess reproducibility, as has been done previously [28]. Hi-C data [9] for IMR90 cells was obtained from the Gene Expression Omnibus (GEO) with accession GSE35156. For both cell types analyses were restricted to reads with alignment mapping quality scores ≥30 and conducted with contact matrices at 25kb resolution since this corresponds to the resolution of SKLLS defined HIRs.

Noised-up versions of simulated chain-like and topologically associated domain (TAD)-like structures and attendant contact maps obtained under differing regimes have been used to evaluate 3D reconstruction algorithms in settings intended to recapitulate practice [34, 42]. Similarly, simulated helical and random walk structures have been used for this purpose [42]. Here we follow an analogous agenda by (i) computing StatDns from the contact matrices provided using each of the normalization schemes described above, and (ii) comparing these to the corresponding structures using *k* nearest neighbors as described subsequently.

As an illustration of how such synthetic data is obtained we present a brief overview of the formulation used for helical structures following Zou et al., [42]. *O*_*ij*_, the (*i*,*j*)^*th*^ entry of the observed contact matrix *O*, is generated as a random Poisson variate with rate parameter *λ*_*ij*_. In turn, this parameter is set using the abovementioned inverse power-law transformation: $\lambda _{ij} = c / d_{ij}^{\alpha }$. Here *d*_*ij*_ corresponds to the distance between the *i*^*th*^ and *j*^*th*^ points on the helix, *α* is fixed at 1.5, and *c* varies so as to govern the signal coverage – the percentage of non-zero entries in the contact matrix. For the results presented subsequently we obtain 100 points on a helix defined by coordinate functions

$$\begin{aligned} x(t) &= 2 \sin(t/3); \ \ \ y(t) = 2 \cos(t/3); \\ z(t) &= t/20; \ \ \ t = 1, \ldots,100. \end{aligned} $$

and set *c* to yield 25% signal coverage, with similar findings at 90% coverage.

### Obtaining 3D genome reconstructions from Hi-C data

Use of simulated 3D architectures and associated contact maps, as above, in evaluating StatDns as a validation tool has the advantage of eliminating uncertainties inherent in the reconstruction process. Nonetheless, it is purposeful to assess StatDns using real data reconstructions, reflecting use in practice.

#### Multi-dimensional scaling

As noted in the Background, there are numerous approaches for generating 3D reconstructions from Hi-C contact maps and, in turn, most of these feature several tuning parameters. In order not to obscure our purpose of appraising StatDns we showcase findings from a simple, minimal-assumption approach to reconstruction: multi-dimensional scaling, fit using the R package smacof [15]. MDS is an established approach to finding configurations that recapitulate dissimilarity measures which, in turn, can be obtained from Hi-C contacts, by power-law transformation for example. Accordingly, MDS-based approaches have been widely used in the context of genome reconstruction [2, 4, 16, 24, 27, 29, 32, 35, 41].

Under MDS we seek a 3D configuration $X = \{\vec {x}_{1},\ldots,\vec {x}_{n}\}; \vec {x}_{j} \in R^{3}$ that best fits the dissimilarity matrix *D* according to:

2 $$ \min_{\{\vec{x}_{1},\ldots,\vec{x}_{n} | \sum \vec{x}_{i} =0 \}} \! \sum_{\{i,j | D_{ij} < \infty\}} \! \omega_{ij} \cdot (\| \vec{x}_{i} - \vec{x}_{j} \| - D_{ij})^{2} \\  $$

Though confining our attention to MDS, we explored a variety of schemes within this framework, using both metric and non-metric scaling, and varying dissimilarity weights *ω*_*ij*_ whereby downweighting of imprecise contact counts can be accommodated, and power-law indices for transforming *O* to *D*. We note that irrespective of MDS reconstruction method examined results were largely similar.

#### Hamiltonian simulated annealing

In order for findings not to be solely reliant on a single (MDS) reconstruction strategy – although, as noted, a range of MDS specifications were examined – we additionally applied the Hamiltonian simulated annealing (HSA, [42]) algorithm. HSA has a number of compelling attributes: (i) it can simultaneously handle multiple data tracks allowing for integration of Hi-C contact data from differing restriction enzyme digests; (ii) it can adaptively estimate the power-law index whereby contacts are transformed to distances, the importance of which has been previously emphasized [41]; and (iii) by using simulated annealing combined with Hamiltonian dynamics it can effectively optimize over for the high dimensional space representing the genomic loci’s 3D coordinates.

Analogous to other 3D reconstruction algorithms [20, 35], HSA models (normalized) contact counts, *n*, via Poisson regression:

3 $$\begin{array}{@{}rcl@{}} n_{i_{k} j_{k}} & \sim & {Poi}(\mu_{i_{k} j_{k}}), \qquad k = 1,\ldots,K  \end{array} $$

4 $$\begin{array}{@{}rcl@{}} \ln(\mu_{i_{k} j_{k}}) & = & \beta_{k0} + \beta_{k1} \ln(d_{i_{k} j_{k}})  \end{array} $$

5 $$\begin{array}{@{}rcl@{}} d_{i_{k} j_{k}} & = & || X_{i_{k}} - X_{j_{k}} ||_{2}  \end{array} $$

where in (3) *k* indexes track and $n_{i_{k} j_{k}}$ is the count for genomic loci *i*_*k*_,*j*_*k*_. The parameters *β*_*k*1_ are (track specific) power-law indices relating expected counts (*μ*) to Euclidean distances (*d*). Covariates such as GC content and fragment length can be included in (4) in order to facilitate in-line normalization. The $X_{i_{k}} = (x_{i_{k}},y_{i_{k}},z_{i_{k}})$ and $X_{j_{k}} = (x_{j_{k}},y_{j_{k}},z_{j_{k}})$ in (5) are the 3D coordinates for loci *i*_*k*_,*j*_*k*_ and constitute the unknown parameters providing the reconstruction. These are subject to constraints designed to capture the local contiguity of chromatin, represented by induced dependencies of a hidden Gaussian Markov chain. The full log-likelihood for *β*,*X* is then

6 $$ \ln(L(\beta,X | \mu, i_{k}, j_{k}) \propto \sum_{k} \sum_{i_{k},j_{k}} \left[ - \exp (\ln(\mu_{i_{k} j_{k}}) + n_{i_{k} j_{k}} (\ln(\mu_{i_{k} j_{k}}))) \right]   $$

to which a penalty term controlling local smoothness is added. Note that (constrained) *X* enters (6) through *μ* and *d* from (4) and (5) respectively. The resulting penalized likelihood is optimized by iterating between generalized linear model (GLM, *cf* Poisson regression) fitting to obtain estimates $\hat \beta $ and simulated annealing to obtain estimates of the 3D coordinates $\hat X = (\hat x, \hat y, \hat z)$. Several tuning parameters control the simulated annealing search and we used default values, as established by the authors’ for their custom R scripts.

### Stationary distribution reproducibility

We assessed the reproducibility – between primary and replicate data series – of StatDns obtained under the differing normalization schemes – using scatterplot smoothing and associated correlations. We contrast these correlations with stratum-adjusted correlation coefficients (SCCs) of the corresponding Hi-C data. SCCs, described below, are custom correlation measures developed for Hi-C contact matrices that reflects the same constant diagonal expected counts described above which, on average, decreases substantially as genomic distance increases [39].

The SCC is based on the generalized Cochran-Mantel-Haenszel statistic, *M*^2^, which is used for testing whether two variables are associated while being stratified by a third variable [1]. Since the magnitude of *M*^2^ depends on sample size it does not provide a direct measure of association strength. In the unstratified setting we have the relationship *ρ*^2^=*M*^2^/(*n*−1) where *ρ* is the Pearson correlation coefficient and *n* is the number of observations. This relationship underscores the derivation of the SCC to measure association in the presence of stratification. Let (*X*,*Y*) denote a pair of samples (here contact matrices) with *n* observations stratified into *K* strata (here diagonal bands corresponding to equal genomic distances), each having *n*_*k*_ observations so that $\sum _{k=1}^{K} n_{k} = n$. Let the observations in stratum *k* be $(x_{i_{k}}, y_{i_{k}}); i = 1, \ldots, K$ with associated random variables (*X*_*k*_,*Y*_*k*_).

The Pearson correlation coefficient *ρ*_*k*_ for the *k*^*th*^ stratum is *ρ*_*k*_=*r*_1*k*_/*r*_2*k*_, where

$${\begin{aligned} r_{1k} & = E(X_{k} Y_{k}) - E(X_{k})E(Y_{k}) \\ & = {{\sum_{i=1}^{n_{k}} x_{i_{k}} y_{i_{k}}} \over{n_{k}}} - {{\sum_{i=1}^{n_{k}} x_{i_{k}} \sum_{j=1}^{n_{k}} y_{j_{k}}} \over {n_{k}^{2}}} \\ r_{2k}^{2} & = Var(X_{k}) Var(Y_{k}) \\ & = \left[{{\sum_{i=1}^{n_{k}} x_{i_{k}}^{2}} \over{n_{k}}} - \left({\sum_{i=1}^{n_{k}} x_{i_{k}}} \over{n_{k}}\right)^{2} \right] \left[{{\sum_{i=1}^{n_{k}} y_{i_{k}}^{2}} \over{n_{k}}} - \left({\sum_{i=1}^{n_{k}} y_{i_{k}}} \over{n_{k}}\right)^{2} \right] \end{aligned}} $$

It is straightforward to represent *M*^2^ in terms of a weighted sum of the *ρ*_*k*_ which gives rise to the SCC defined as

7 $$ \rho_{s} = \sum_{k=1}^{K} \left({n_{k} r_{2k}} \over {\sum_{k=1}^{K} n_{k} r_{2k}} \right) \rho_{k}.  $$

Further aspects of SCCs, including obtaining the variance of *ρ*_*s*_, deploying variance stabilizing weights in computing *ρ*_*s*_, guidelines for determining the number of strata *K* are detailed in Yang et al., [39], with fitting making recourse to R package hicrep [40].

### Comparing stationary distributions and 3D genome reconstructions

For each locus of a 3D structure, either simulated or obtained via reconstruction, we compute the distance to its *k*^*th*^ nearest neighbor (*k*NN) in the structure, for *k*∈*Ω*={5,15,25}, using the R package FNN [5]. Since *k*NN distances are monotone in *k* it suffices to consider a few select values. We plot these *k*NN distances against StatDn values obtained from the corresponding contact matrix. We again use scatterplot smoothing (R function lowess) to highlight relationships, with a monotone decreasing association anticipated if StatDn identification of highly (and remotely) interacting loci are supported by the structure. To appreciate the basis for this monotone decreasing relationship consider the antithesis of a HIR, namely a minimally interacting region, characterized by low StatDn values. By virtue of its minimal interactions nearest neighbor distances for given *k*∈*Ω* will be large. The converse holds for HIRs and the underlying high StatDn values leading to the monotone decreasing relationship between StatDns and *k*NN distances.

## Results

Our findings are presented largely by way of figures. These are constructed so that comparisons between *O*,*O*−*E*,*O*/*E* normalizations are highlighted. But, more important than these internal contrasts are overall assessments of StatDns for the stated objective of appraising 3D reconstructions. In most of the settings considered the overall performance is such that StatDns cannot be endorsed as a 3D reconstruction evaluation technique since the abovementioned monotone decreasing relationship with *k*NN distances fails to hold. Moreover, examples wherein anomalous behavior of StatDns is exhibited are showcased.

We report results for GM12878 chromosome 9 since this exhibits the highest density (per base) of HIRs as defined by SKLLS. We also present results for GM12878 chromosome 4 which is relatively sparse with respect to HIRs. However, similar trends were consistently observed across all chromosomes examined (not shown). Additionally, findings from select IMR90 cells are illustrated, revealing instances of StatDn breakdown.

### Stationary distribution reproducibility

**Fig. 1:**
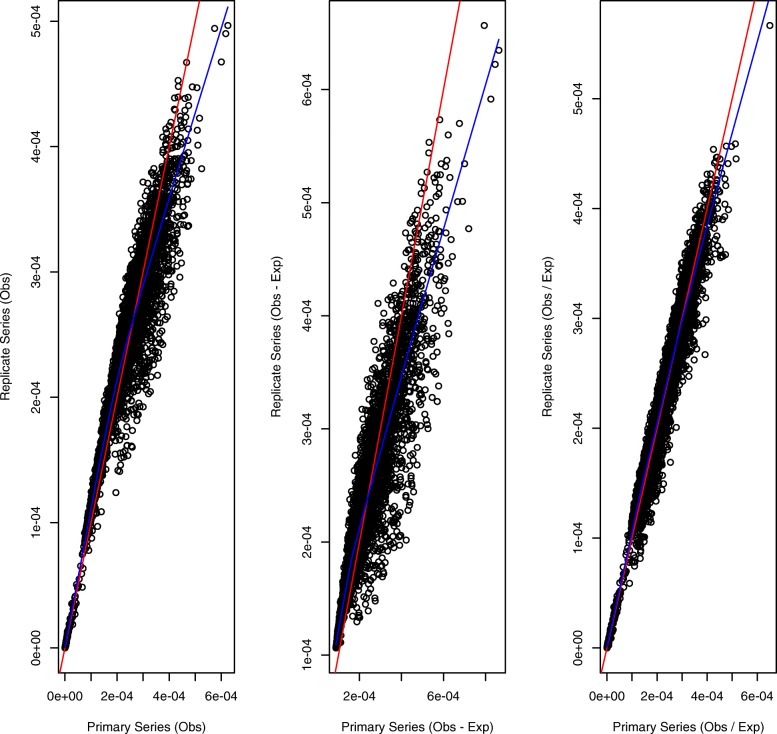
StatDn reproducibility for GM12878 Chromosome 9. Agreement between Stationary Distributions obtained from primary and replicate series Hi-C data at 25kb resolution [23]. StatDn normalization schemes are *O* (left panel), *O*−*E* (middle) and *O*/*E* (right). In each panel the identity line is in red and the lowess smooth is in blue

In Fig. 1 we compare the StatDns of GM12878 cells chromosome 9 primary and replicate series corresponding to respective normalizations *O*,*O*−*E*,*O*/*E*. The respective correlations are 0.962, 0.937 and 0.977 whereas the SCC between the primary and replicate contact matrices is 0.966. Thus, reproducibility for the *O*−*E* normalization chosen by SKLLS is furthest removed from the correlation between the underlying contact matrices.

**Fig. 2:**
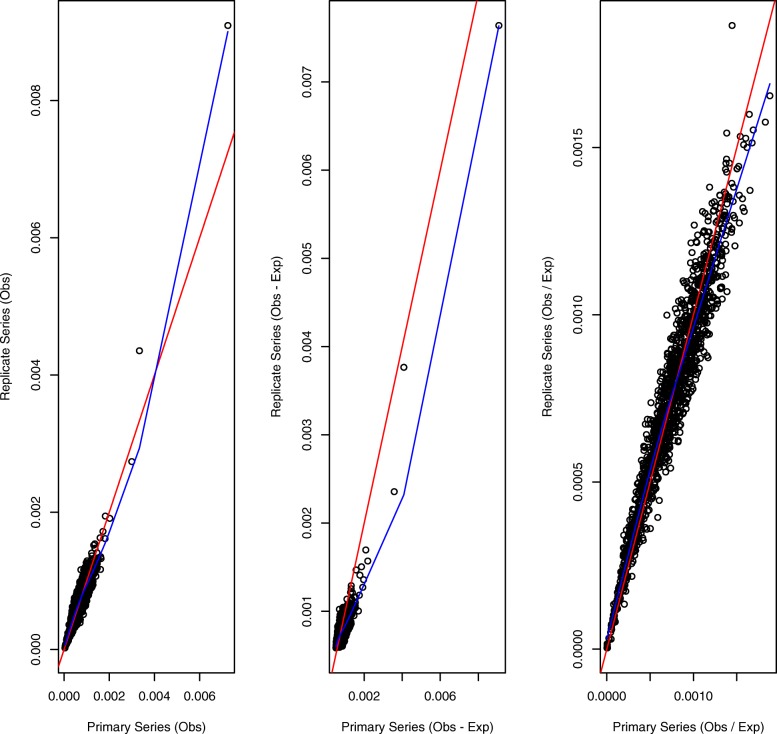
StatDn reproducibility for IMR90 chromosome 21. Agreement between Stationary Distributions obtained from primary and replicate series Hi-C data at 25kb resolution [9]. StatDn normalization schemes are *O* (left panel), *O*−*E* (middle) and *O*/*E* (right). In each panel the identity line is in red and the lowess smooth is in blue

More interesting findings emerge when we similarly assess reproducibility for IMR90 cells. Figure 2 displays the StatDns for IMR90 chromosome 21 primary and replicate series, again corresponding to respective normalizations *O*,*O*−*E*,*O*/*E*. The corresponding correlations are 0.935, 0.936 and 0.966, whereas the SCC between the primary and replicate contact matrices is 0.808. Thus, the StatDn correlations appreciably exceed the SCC between the underlying contact matrices, indicative of possible problems with StatDns in view of the careful and contact map customized construction of SCCs [39].

Also apparent in Fig. 2 are StatDn outliers, for both *O* and the chosen *O*−*E* normalizations, which result from (relatively) extreme contact matrix row sums, indicating possible normalization breakdown for such instances. An even more dramatic example of anomalous StatDn values is shown below with respect to reconstruction (Fig. 8).

### Relating stationary distributions to 3D structures

The simulated helical and random walk structures previously used for 3D reconstruction evaluation [42] include instances varying according to the extent of signal coverage, defined as the percentage of non-zero entries in the contact matrix derived from the generated structure. Here we illustrate results for the lowest levels of signal coverage: 25% and 10% for the helix and random walk respectively. Findings at higher levels of signal coverage are similar (not shown) although the helical structure with 90% signal coverage does not display a monotone decreasing relationship between *k*NN distances and StatDns with *O*/*E* normalization.

**Fig. 3:**
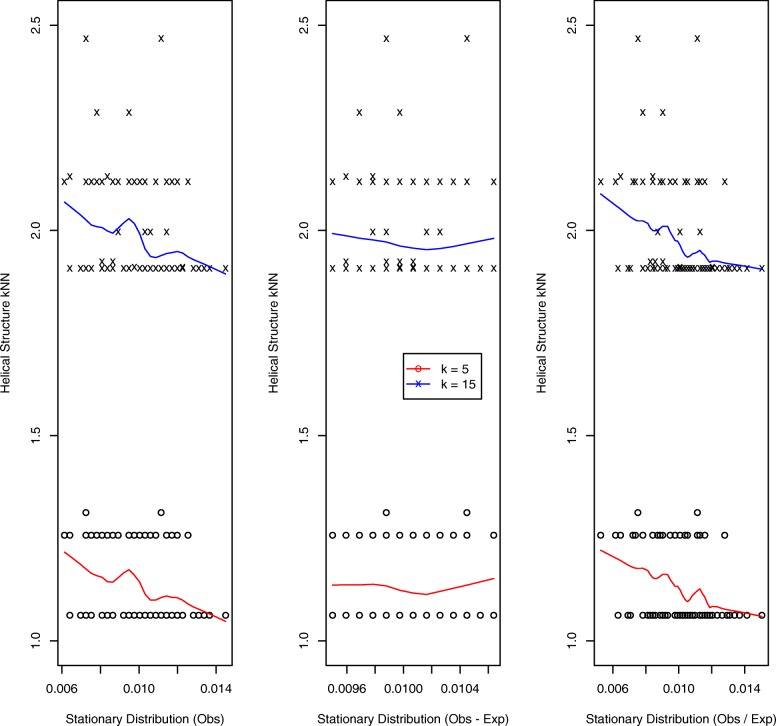
Helical structure: *k*NNs versus StatDns. Relationships between *k* nearest neighbors and StatDns for *k*=5 (o, red lowess smooth) and *k*=15 (x, blue lowess smooth) for the simulated helical structure generated to have 25% signal coverage (percentage of non-zero contact matrix entries) per [42]. StatDn normalization schemes are *O* (left panel), *O*−*E* (middle) and *O*/*E* (right)

**Fig. 4:**
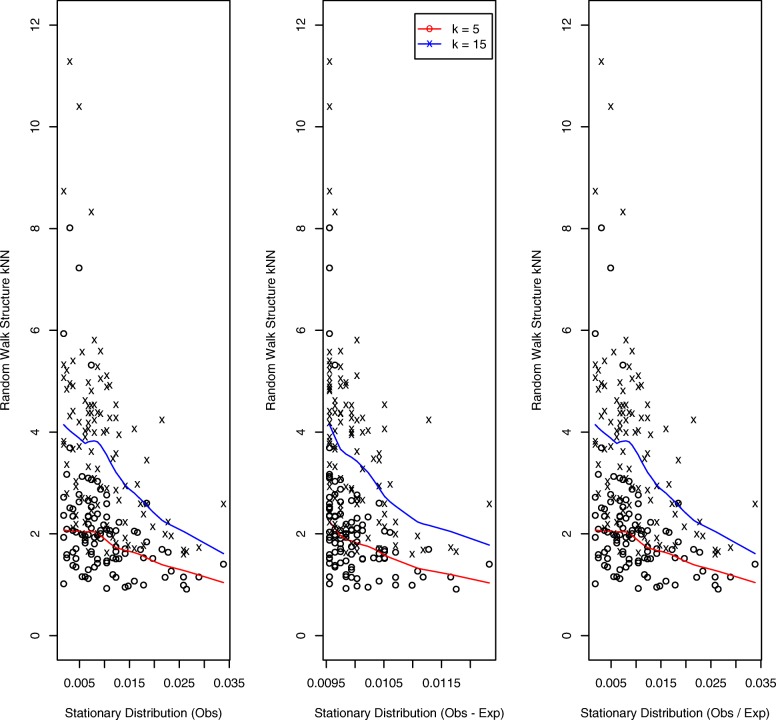
Random walk structure: *k*NNs versus StatDns. As for Figure 3 but for the simulated random walk structure generated to have 10% signal coverage per [42]

Results for the simulated helical structure, based on 100 loci, are presented in Fig. 3. The quantal nature of the *k*NN distances (we display results for *k*=5,15) – for example, there are only three distinct 5 nearest neighbor distances – reflects the regularity of the helical configuration. The left and right panels, corresponding to *O* and *O*/*E* normalization, exhibit decreasing trends: the higher the StatDn value, nominally corresponding to loci with greater numbers of interactions, the smaller the *k*NN distance in the structure, as would be expected. However, for the middle panel, corresponding to *O*−*E* normalization, no such relationship is evident. Further, by virtue of the manner whereby *O*−*E* normalization handles non-positive values, there is substantial duplication of StatDn values: 47 uniques versus 97 for *O*,*O*/*E*. Results for the random walk structure are presented in Fig. 4. Here we see very similar performance across normalization schemes with the anticipated decreasing relationship exhibited for each.

**Fig. 5:**
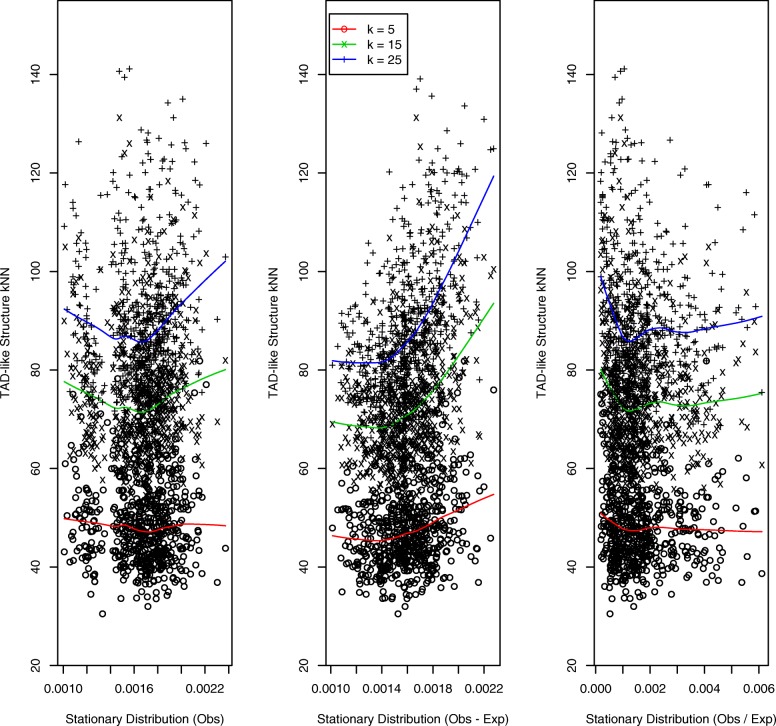
TAD-like structure: *k*NNs versus StatDns. As for Fig. 3 but for the simulated TAD-like structure generated to have mid-level noise and structural variability (*α*=100 and *Δt*=10^3^) per [34] and with *k*NNs: *k*=5 (o, red lowess smooth), *k*=15 (x, green lowess smooth) and *k*=25 (+, blue lowess smooth)

**Fig. 6:**
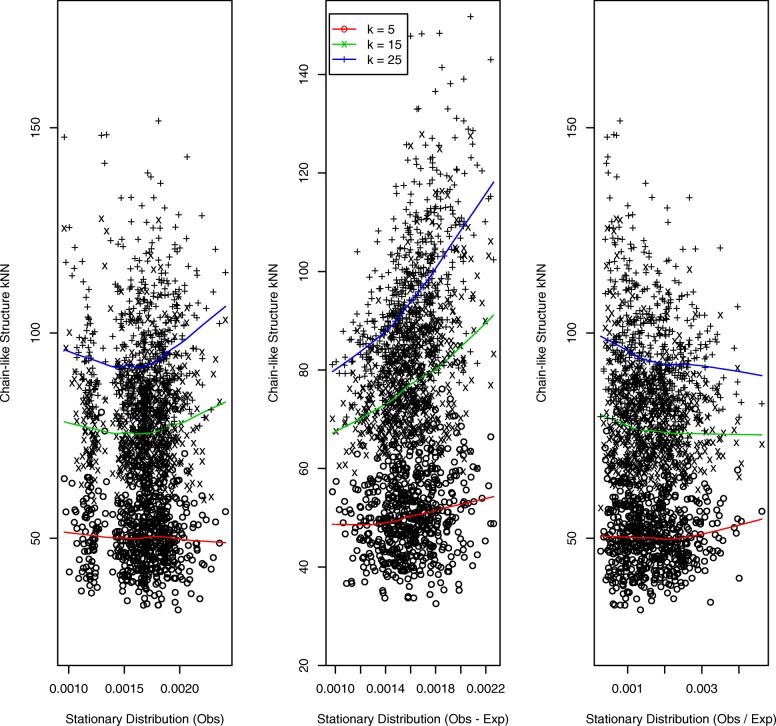
Chain-like structure: *k*NNs versus StatDns. As for Fig. 5 but for the simulated chain-like structure

A comprehensive effort to generate structures and attendant contact matrices that more realistically reflect chromatin architecture has been undertaken by Trussart et al., [34]. Here we focus on two such structures, TAD-like and chain-like, each generated with mid-level noise and structural variability corresponding to Trussart et al., parameter settings of *α*=100 and *Δt*=10^3^ respectively. Results for the TAD-like structure are presented in Fig. 5 and for the chain-like structure in Fig. 6. For both structures we observe StatDns displaying an *increasing* relationship with *k*NN distances, this being strongest for *O*−*E* normalization.

**Fig. 7:**
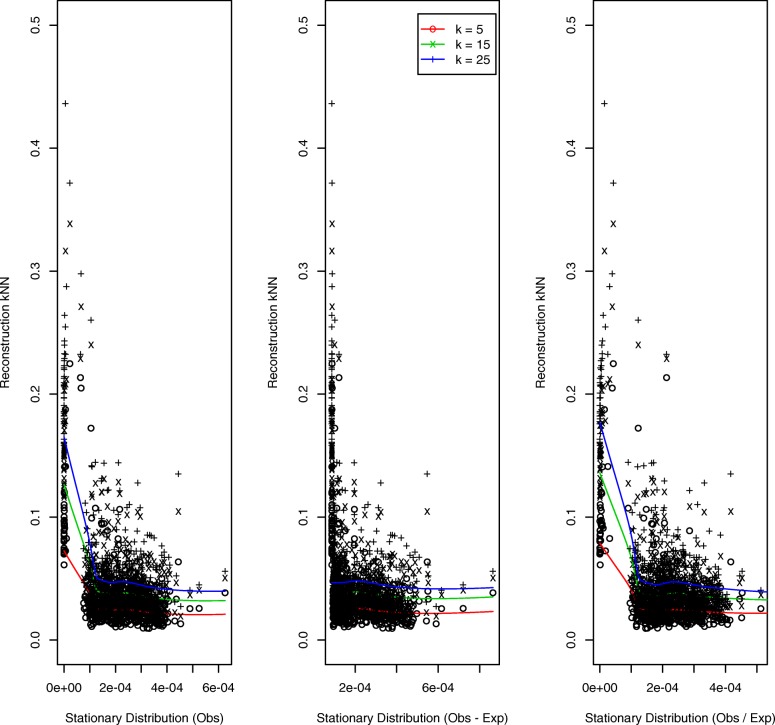
GM12878 Chromosome 9: *k*NNs versus StatDns. As for Fig. 5 but for reconstructed GM12878 Chromosome 9 where the reconstruction utilized unweighted metric MDS. While plotted points correspond to 500 randomly sampled loci (≈ 10% of the total), the depicted lowess smooths are based on the entire sample

**Fig. 8:**
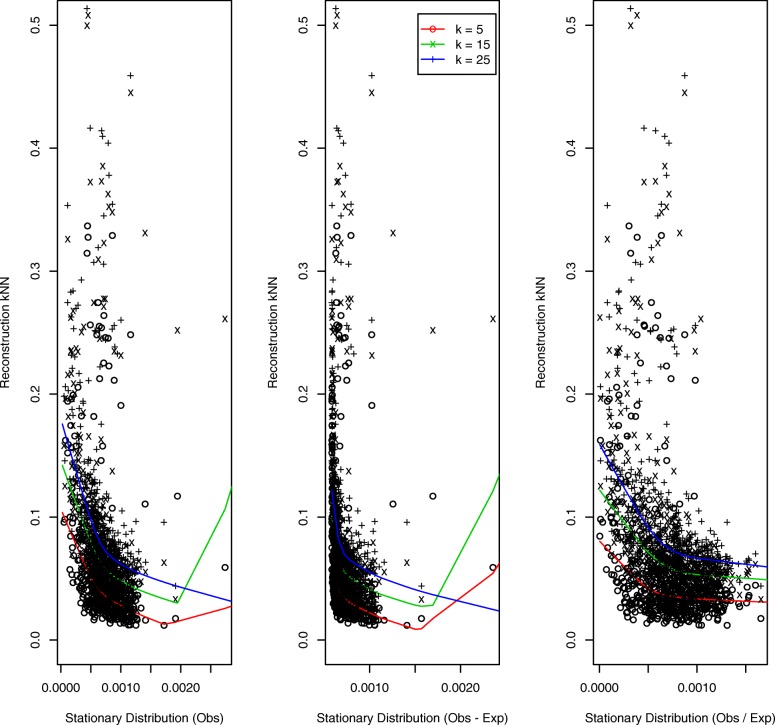
IMR90 Chromosome 21: *k*NNs versus StatDns. As for Fig. 7 but for reconstructed IMR90 Chromosome 21 where the reconstruction utilized HSA. While plotted points correspond to 500 randomly sampled loci (≈ 35% of the total), the depicted lowess smooths are based on the entire sample

Results from StatDn evaluation of a reconstruction for GM12878 chromosome 9 via unweighted metric MDS are depicted in Fig. 7. While the left and right panels corresponding to *O* and *O*/*E* normalization display decreasing relationships with *k*NN distances these are driven by elevated *k*NN values for small StatDn probabilities. Results for *O*−*E* normalization are effectively constant. Analogous findings were obtained from other (weighted, non-metric) MDS reconstruction approaches, as well as for HSA-based reconstruction.

Similarly, results from StatDn evaluation of a reconstruction for IMR90 chromosome 21 by HSA are depicted in Fig. 8. Here the left and middle panels corresponding to *O* and *O*−*E* normalization display decreasing relationships with *k*NN for the bulk of the data but exhibit increasing trends in the upper tail: the region containing the HIR. These same trends were evident in reconstructions obtained using MDS.

## Discussion

Many potential difficulties surrounding use of StatDns were delineated in Methods under Normalization and Interpretation Issues and these concerns have been borne out by the empirical results. It is important to note that these problems cannot be ascribed to deficiencies of the reconstruction algorithms since they are also exhibited with simulated structures that bypass the reconstruction step. Moreover, for some of the explorations based on chromatin configuration reconstruction, we have deliberately opted to utilize a minimalist MDS approach, thereby limiting the influence of assumptions and parameter tuning. These findings, wherein StatDns do not recapitulate inferred 3D MDS reconstructions, also pertain to an alternate state-of-the-art reconstruction algorithm, HSA, and hold across all cell lines and chromosomes examined. Thus, the overall weight of evidence, both theoretic and empirical, is such that StatDns, especially those based on the prescribed *O*−*E* normalization, cannot be recommended as a means for evaluating 3D genome reconstruction. Indeed, these problematic underpinnings of StatDns, including the logic surrounding their definition, call into question their usage for any purpose, not just reconstruction assessment as examined here.

This conclusion begs the question as to whether alternate, established structural units derived from Hi-C contact matrices, such as TADs [9] and contact domains [23], might serve as components for (non-orthogonal) reconstruction assessment. However, these constructs are by definition local and so do not provide a basis for effecting large-scale structure interrogation. It was the purported ability of StatDns to capture frequent, long-range interactions that motivated this evaluation of their validation potential. Conversely, TADs [24] and FISH distances [29] have been used to improve the reconstruction process itself. Again, given their uncertain foundation, we see no analogous role for StatDns.

## Conclusion

Our analyses demonstrate that, as constructed, StatDns do *not* provide a suitable measure for assessing the accuracy of 3D genome reconstructions. Whether this is attributable to specific choices surrounding their formulation or to the logic underlying their very definition remains to be determined.

## Data Availability

Hi-C data for GM12878 cells is available from GEO with accession GSE63525: https://www.ncbi.nlm.nih.gov/geo/query/acc.cgi?acc=GSE63525. Hi-C data for IMR90 cells is available from GEO with accession GSE35156: https://www.ncbi.nlm.nih.gov/geo/query/acc.cgi?acc=GSE35156. Contact maps and associated structures corresponding to chain-like and TAD-like models [34] were obtained from http://sgt.cnag.cat/3dg/datasets/. The noised-up helical (regular) and random walk structures and attendant contact matrices utilized in [42] are available from https://people.umass.edu/ouyanglab/hsa/downloads.html#Data.

## Abbreviations

3D: Three dimensional
FISH: Fluorescence in situ hybridization
GEO: Gene expression Omnibus
HIRs: Highly interactive regions
HSA: Hamiltonian simulated annealing
*k*NNs: *k* Nearest neighbors
MDS: Multi-dimensional scaling
SCC: Stratified correlation coefficient
SKLLS: Sobhy, Kumar, Lewerentz, Lizana, Stenberg
StatDn: Stationary distribution
TAD: Topologically associated domain
TPM: Transition probability matrix
